# Importance of preovulatory estradiol on uterine receptivity and luteal function

**DOI:** 10.1590/1984-3143-AR2023-0061

**Published:** 2023-09-04

**Authors:** George Allen Perry, Jaclyn Nicole Ketchum, Lacey Kay Quail

**Affiliations:** 1 Texas A&M AgriLife Research, Overton, Texas, United States of America; 2 Department of Animal Science, Texas A&M University, College Station, Texas, United States of America

**Keywords:** estradiol, uterus, corpus luteum, oviduct

## Abstract

Animals that exhibited estrus had greater pregnancy success compared to animals that did not exhibit estrus before fixed-time AI (FTAI). Estradiol is synthesized in bovine ovarian follicles under gonadotropin regulation and can directly and indirectly regulate the uterine receptivity and luteal function. Estradiol concentrations at FTAI impacted oviductal gene expression and has been reported to play an important role in establishing the timing of uterine receptivity. These changes have been reported to impact uterine pH and sperm transport to the site of fertilization. After fertilization, preovulatory estradiol has been reported to improve embryo survival likely by mediating changes in uterine blood flow, endometrial thickness and changes in histotroph. Cows with greater estradiol concentrations at the time of GnRH-induced ovulation also had a larger dominant follicle size and greater circulating progesterone concentrations on day 7. Therefore, it is impossible to accurately determine the individual benefit of greater estradiol concentrations prior to ovulation and greater progesterone concentrations following ovulation to pregnancy establishment, as these two measurements are confounded. Research has indicated an importance in the occurrence and timing of increasing preovulatory concentrations of estradiol, but increasing estradiol concentrations by supplementation may not be sufficient to increase fertility. Increased production of estradiol by the preovulatory follicle may be required to enhance fertility through the regulation of sperm transport, fertilization, oviductal secretions, the uterine environment, and embryo survival.

## Introduction

Estrous synchronization and AI remain an important and widely applicable reproductive biotechnology available for cattle (Seidel, 1995). However, time and labor deter its widespread utilization. With development of FTAI protocols, estrus detection is not necessary as a gonadotropin-releasing hormone (GnRH) agonist can cause ovulation (Ryan et al., 1998). Nevertheless, successful implementation of a FTAI protocol requires a large proportion of females to express estrus, as a positive relationship between estrus expression at time of insemination and pregnancy success has been established in both beef and dairy cattle (Vasconcelos et al., 2001; Perry et al., 2005; Lopes et al., 2007; Perry et al., 2007). Animals that exhibited estrus averaged 27% greater pregnancy success compared to animals that did not (Richardson et al., 2016). Furthermore, the technology of embryo transfer provides the opportunity to increase the quantity of genetically superior offspring in a shorter period of time (Lohuis, 1995; Hasler, 2014); however, for this technology to be successful, recipient females need to express estrus as pregnancy per embryo transfer (P/ET) are greater among estrual recipients (Baruselli et al., 2003; Bó and Cedeño, 2018).

Estradiol is synthesized in bovine ovarian follicles under gonadotropin regulation, described by the two cell-two gonadotropin model (Fortune and Quirk, 1988). Preovulatory estradiol has several physiological roles in pregnancy establishment, including the expression of estrus (Coe and Allrich, 1989), induction of the preovulatory gonadotropin surge (Chenault et al., 1975), facilitating the transport of sperm (Hawk and Cooper, 1975), and inducing endometrial progesterone receptors (Zelinski et al., 1982).

## The role of estradiol in regulating fertility

### Estrus

Estrus refers the time period when a female is sexually receptive. Standing estrus refers to the behavioral response of a female standing to be mounted by a male or another female. Initiation of estrus occurs due to increased circulating concentrations of estradiol when progesterone concentrations are low (Allrich, 1994). Among cattle, concentrations of estradiol peak approximately 36 hours before ovulation (Chenault et al., 1975), and increased preovulatory concentrations of estradiol have been correlated with increased pregnancy success (Perry et al., 2005). Furthermore, estrus expression, when compared to no estrus expression, reduced pregnancy loss from day 32 to day 60 following FTAI (Pereira et al., 2014). While expression of estrus contributes to the establishment of pregnancy, there has been no repeatability of expression of estrus reported (Richardson et al., 2016).

### Oviduct

The oviductal environment is unique and governed by steroid hormones throughout the estrous cycle. Lipid and enzyme production by the oviductal epithelium increases in response to estradiol (Witkowska, 1979), and oviductal fluid during estrus also contains a sperm capacitation factor (Parrish et al., 1989). Perhaps most notably, oviductal glycoprotein secretion is maximized at estrus (Stanke et al., 1974; Malayer et al., 1988) in response to estradiol associating with its stromal receptor (Nancarrow and Hill, 1994). Estrus-associated glycoprotein is produced by both the ampulla and isthmus regions of the bovine oviduct (Boice et al., 1990), and interacts with spermatozoa (King and Killian, 1994) and oocytes (Wegner and Killian, 1991) in such a way that improves fertilization, cleavage rates, and blastocyst formation in a dose-dependent manner (Hill et al., 1996; Martus et al., 1998). Additionally, when cyclic ewes were ovariectomized, production of estrus-associated glycoprotein was abolished, but administration of estradiol benzoate (EB) restored estrus-associated glycoprotein production (Sutton et al., 1986).

In addition to changes in oviductal fluid, oviductal gene expression is different across the estrous cycle, such that expression of 37 genes related to protein secretion and modification were upregulated during estrus compared to diestrus in the oviduct of heifers (Bauersachs et al., 2003). More specifically, expression of Microsomal Prostaglandin E Synthase-1 (MPGES-1) mRNA, a member of the prostaglandin family known for regulation of ovulation, fertilization, and implantation (Lim et al., 1997) as well as oviductal contraction (Wijayagunawardane et al., 2001), was greater during periods of estrogen dominance compared to progesterone dominance in all regions of the bovine oviduct (Gauvreau et al., 2010). Additionally, loss of the oviductal estrogen receptor increases protease activity, which subsequently results in embryonic mortality within the first two days of pregnancy (Winuthayanon et al., 2015).

Estradiol concentrations at FTAI impact oviductal gene expression, such that 1386 and 61 genes were up- and down-regulated, respectively, at the ampullary-isthmic junction, while 349 and 202 genes were up- and down-regulated, respectively, in the isthmus of cows with increased circulating estradiol concentrations (Quail et al., 2021). More specifically, KEGG pathways associated with metabolism and hormone signaling were associated with up-regulated genes at the ampullary-isthmus junction, while up-regulated genes in the isthmus were associated with the cell adhesion KEGG pathway in cows with increased estradiol concentrations at FTAI (Quail et al., 2021).

### Uterine environment

Estradiol has been reported to play an important role in establishing the timing of uterine receptivity (Ozturk and Demir, 2010). The uterine environment is necessary to fertilization, early embryo development, recognition of pregnancy, as well as conceptus elongation and attachment. During the estrous cycle, endometrial changes in composition and differentiation are regulated by estradiol, progesterone, and oxytocin (Spencer and Bazer, 2004), and the timing of these changes is critical to embryo survival. Thus, estradiol plays an important role in establishing the timing of uterine receptivity (Ozturk and Demir, 2010). In cattle, synchrony between the embryo and uterus must be ± 24 hours (Hasler, 2001).

#### Uterine gene expression

A study conducted by Zelinski et al. (1982) concluded that estradiol induces synthesis of endometrial cytoplasmic estrogen and progesterone receptors (Zelinski et al., 1982), which further supports work from (Koligian and Stormshak, 1977) concluding progesterone inhibited the replenishment of cytoplasmic estradiol receptors during the luteal phase in the ovine endometrium. More recently, it has been reported that progesterone receptors in the deep glandular epithelium, as well as endometrial estradiol receptor (ERα) mRNA, were up-regulated on day 15.5 in cows with elevated preovulatory estradiol concentrations (Bridges et al., 2012).

Differences also exist in endometrial and corpus luteum (CL) gene expression between estrual and nonestrual females, with endometrial transcripts related to prostaglandin synthesis (OTR and COX-2) as well as the immune system and cell adhesion (CXCL10, IGLL1, MX1, MX2, MMP19, MYL12A, and SLPI) influenced by the expression of estrus (Davoodi et al., 2016). Furthermore, the abundance of facilitative and sodium-dependent glucose transporters, which are responsible for altering the composition of uterine luminal fluid (ULF) and providing glucose to the developing conceptus, is impacted by estradiol, such that females with elevated preovulatory estradiol concentrations had a greater abundance of SLC2A1 and SLC5A1 in both intercaruncular and caruncular tissues (Northrop et al., 2018).

The TGF-β superfamily is involved in endometrial changes, placental development, and pregnancy maintenance (Jones et al., 2006). At estrus, there is also up-regulation of several genes involved in remodeling the extracellular matrix (Bauersachs et al., 2005), as well as changes in expression of inhibin A subunit (a member of the TGF-β signaling pathway) in the bovine intercaruncular area, suggesting the involvement of estradiol in coordinating endometrial remodeling (Ishiwata et al., 2003). Milk protein (SERPINA14) is expressed in the endometrium of ruminants during pregnancy, and is likely involved in nutrition of the embryo/fetus, embryonic/fetal growth, and suppression of the maternal immune system (Ing and Roberts, 1989). Expression of SERPINA14, as determined by Real-time RT-PCR, was greatest on the day of estrus, and was also up-regulated after stimulation with estradiol. Furthermore, detection of the SERPINA14 protein revealed the protein was localized to the glandular epithelium and was increased on the day of estrus (Ulbrich et al., 2009). This suggests a possible preparatory role for preovulatory estradiol in establishing a uterine environment that is conducive to pregnancy.

CLOCK genes regulate biological oscillations and the transcription of other genes in a tissue specific manner in response to changes in nutrient status, day length, and possibly other environmental influences (Albrecht, 2006). Decreased litter size, lower frequency of successful matings, and increased cycle length have been reported in ClockΔ19 (mice in which CLOCK is not functional) female mice (Chappell et al., 2003). When ClockΔ19 female mice were treated hormonally to induce ovulation and mated with fertile males, ClockΔ19 females produced fewer embryos than control females (Kennaway, 2005). From these results, it is possible that circadian clock gene defects at the level of the uterus may disrupt the synchrony between the uterine environment and the developing embryo. He et al. (2007) reported that Period 1 mRNA (part of the positive feedback loop of CLOCK) was expressed in the uterus of diestrus rats, and that both estradiol and progesterone stimulated uterine Period 1 mRNA expression in ovariectomized rats. Furthermore, Nakamura et al. (2005) reported that estradiol altered the normal circadian rhythms in the uterus. Therefore, the circadian clock genes may influence fertility at the uterine level.

#### Uterine pH

In estrual females, there was a transient decline in uterine pH from 36 hours prior to the onset of estrus until estrus. From the onset of estrus to approximately 6 hours after the onset of estrus, uterine pH increased rapidly. Neither the decrease in uterine pH prior to estrus, nor the rise after estrus was observed in females that did not express estrus (Perry and Perry, 2008a). Overall, females expressing estrus had a decreased uterine pH (pH = 6.72) compared to females that did not express estrus (pH = 7.0). The effect of preovulatory estradiol on uterine pH was evaluated in females that were or were not treated with 1 mg of estradiol cypionate (ECP) 36 hours prior to the final administration of GnRH in a CO-Synch protocol. Nonestrual females that did not receive ECP had an elevated uterine pH (7.0 ± 0.07) compared to estrual females that were not administered ECP (pH = 6.72 ± 0.10; P = 0.02) and nonestrual females that were administered ECP (6.81 ± 0.09; P = 0.06; Perry and Perry, 2008b). Bolzenius et al. (2016) reported that as uterine pH decreased at the time of FTAI, pregnancy rates increased. They also noted that NA+/H+ exchanger isoforms 1, 2, and 3 played a role in altering uterine pH during the onset of estrus (Bolzenius et al., 2016). Given that Jones and Bavister (2000) reported that as pH decreased, motility of bull sperm decreased and longevity increased, a transient change in uterine pH around estrus may provide a mechanism for increasing sperm longevity in the reproductive tract.

Hawk (1983) reported that in order for sperm to be efficiently transported in the female reproductive tract, females needed exposure to estradiol. At initiation of estrus, uterine pH was decreased which may have led to greater longevity of sperm through a transient decrease in motility (Wong et al., 1981). The peak in uterine pH observed 6 hours after estrus may play a role in aiding sperm transport, as Goltz et al., (1988) reported as pH increased, so did sperm motility (Goltz et al., 1988). Cows that initiated standing estrus had decreased uterine pH (6.78) and increased pregnancy success (52%) compared to cows that did not initiate standing estrus, but were induced to ovulate (6.96 and 38%, respectively). This increase in pregnancy success among cows that exhibited standing estrus is likely due to increased sperm transport to the site of fertilization (Larimore et al., 2015). In a FTAI scenario, the second administration of GnRH can induce ovulation approximately 30 hours after administration in the absence of elevated estradiol concentrations (Pursley et al., 1995; Vasconcelos et al., 1999), and sperm survival until ovulation may be compromised as the interval from insemination to ovulation is lengthened.

#### Fertilization

A review by Santos et al. (2004) reported fertilization failure in lactating beef and dairy cows was as high as 45%. A study in beef cows, where embryos were flushed from cows with high or low estradiol concentrations at the time of FTAI, revealed that cows with greater concentrations of estradiol at GnRH-induced ovulation were more likely to yield a fertilized embryo than an unfertilized oocyte (Jinks et al., 2013). Additionally, animals that exhibited estrus prior to FTAI had increased accessory sperm numbers and improved embryo quality compared to animals that did not exhibit estrus (Larimore et al., 2015). Although accessory sperm are not involved in fertilization, they represent sperm that were able to access the oviduct, undergo capacitation and the acrosome reaction, recognize and bind to the oocyte, and partially penetrate the zona pellucida (Dalton et al., 2006). The number of accessory sperm trapped in the zona pellucida has been positively associated with fertility (Hunter and Wilmut, 1984; Hawk and Tanabe, 1986; DeJarnette et al., 1992; Nadir et al., 1993), and are thought to be an indirect measure of both sperm transport and the availability of competent sperm competing for fertilization (DeJarnette et al., 1992).

#### Uterine blood flow

Endocrine, paracrine, and autocrine factors are all involved in the development and growth of the embryo and placenta. The bovine fetus receives nutrients through both hematotroph and histotroph. Hematotroph allows exchange of nutrients between fetal and maternal circulation (Bazer et al., 1990). When transrectal doppler ultrasonography was used to characterize uterine blood flow, circulating concentrations of estradiol were determined to be greatest on day -2 (day 0 = ovulation) and uterine blood flow was greatest on day -3 (Bollwein et al., 2000). Similarly, a positive correlation between circulating concentrations of estradiol and uterine blood flow as well as uterine artery diameter were determined following administration of EB (10 mg; Rawy et al., 2018). Blood flow increased two days prior to estrus, and remained elevated until the day after estrus, which corresponded with elevated estradiol concentrations (Ford and Chenault, 1981).

Uterine blood flow was similar in pregnant and nonpregnant cows up to 13 days post-mating, at which time blood flow to the gravid horn increased. From day 25 to day 30, blood flow increased to the gravid horn and decreased to the nongravid horn. Progesterone concentrations and uterine blood flow to the gravid horn was positively correlated. The increase in blood flow to the gravid horn, caused by the preimplantation conceptus, was similar to blood flow observed when estradiol was elevated (Ford and Chenault, 1981). The increase in blood flow may also increase the blood flow to the CL found on the ipsilateral ovary, thereby increasing progesterone secretion which would aid in maintaining pregnancy (Ford and Chenault, 1981).

#### Uterine endometrial thickness

Sugiura et al. (2018) reported that as progesterone concentrations decrease, endometrial thickness increased; however, estradiol may also play a role in sustaining and/or enhancing these changes, as endometrial thickness was strongly correlated with the Estradiol:Progesterone ratio after natural and induced estrus (Sugiura et al., 2018). Endometrial thickness was measured by transrectal ultrasonography in lactating Holstein cows administered the Ovsynch protocol. Thickness of the endometrium increased from 7 to 9.5 mm following prostaglandin administration, remained thick (> 9 mm) for two days, and then became thinner on both day 1 (8 mm) and 2 (7.4 mm) following the second administration of GnRH. Supplementing estradiol-17β (1mg) eight hours prior to the second administration of GnRH increased pregnancies to AI in females with thinner endometrium. However, in females that had an endometrial thickness measurement of > 8 mm 48 hours following prostaglandin administration, estradiol supplementation did not improve pregnancies to AI (Souza et al., 2011).

#### Histotroph

Uterine histotroph is composed of nutrients, growth factors, proteins, glucose, immunosuppressive agents, enzymes, and ions. It is secreted by the endometrium and is necessary for early conceptus growth/survival (Geisert et al., 1992; Gray et al., 2001). Any changes can greatly influence early embryonic viability. In particular, glucose is a major fuel source used by the conceptus for growth and development. Animals that exhibited estrus during a FTAI protocol had greater glucose concentrations in the ULF compared to nonestrual animals (Northrop et al., 2018). When administration of estrogen, corresponding with initiation of estrus, was omitted in ovariectomized, hormone-supplemented ewes, embryo survival following embryo transfer (Miller and Moore, 1976), uterine weight, uterine protein, RNA to DNA ratio, and the rate of protein synthesis were decreased (Miller et al., 1977).

Overall, cows that exhibited standing estrus around the time of FTAI had increased preovulatory concentrations of estradiol (Perry et al., 2005; Perry and Perry, 2008a; Perry and Perry, 2008b), decreased uterine pH (Perry and Perry, 2008a; Perry and Perry, 2008b), increased sperm transport (Larimore et al., 2015), and increased pregnancy success (Perry et al., 2005) compared to nonestrual cows. Consequently, preovulatory concentrations of estradiol may play a major role in the uterine environment as well as the establishment and maintenance of pregnancy.

### Subsequent progesterone effects

Two of the most important factors involved in the establishment of pregnancy are the circulating estradiol concentration preceding GnRH-induced ovulation and circulating progesterone concentration on day 7 following ovulation (Atkins et al., 2013; Jinks et al., 2013). However, cows with greater estradiol concentrations at the time of GnRH-induced ovulation also have a larger dominant follicle size and greater circulating progesterone concentrations on day 7 (Jinks et al., 2013). Among dairy cows, cows that ovulated small follicles (10 to 15 mm) had decreased concentrations of progesterone on day 7 (Sartori et al., 2006) and cows that were AIed with decreased concentrations of progesterone on day 7 had decreased P/AI, but when embryos were transferred there was no relationship between concentrations of progesterone and P/ET (Sartori et al., 2006; Demetrio et al., 2007). Therefore, the direct impact of increase preovulatory estradiol likely occurs in the oocyte during final maturation or in early embryo development before day 7.

The preovulatory follicular environment is important for preparing follicular cells for luteinization and secretion of progesterone (McNatty et al., 1975). McNatty et al. (1979) suggested that development of a normal CL depends on a follicle meeting the following criteria: 1) an adequate number of granulosa cells, 2) an adequate number of luteinizing hormone (LH) receptors on the granulosa and theca cells, and 3) granulosa cells capable of synthesizing adequate amounts of progesterone following luteinization. Within granulosa cells, estradiol is reported to cause: 1) increased cellular proliferation (Goldenberg et al., 1972; Parrott and Skinner, 1998; Dupont et al., 2000), 2) formation of gap junctions (Merk et al., 1972; Burghardt and Anderson, 1981), 3) increased stimulatory action of follicle-stimulating hormone (FSH) on aromatase activity (Adashi and Hsueh, 1982; Zhuang et al., 1982; Reilly et al., 1996), 4) enhanced stimulation of progesterone synthesis following gonadotropin stimulation (Welsh et al., 1983; Fanjul et al., 1984), and 5) enhanced acquisition of LH receptors (Kessel et al., 1985; Farookhi and Desjardins, 1986; Wang and Greenwald, 1993). Furthermore, luteinized granulosa cells secreted increased progesterone when they were collected from follicles having increased estradiol concentrations compared to granulosa cells from follicles that had decreased estradiol concentrations (McNatty et al., 1979).

The relationship between pregnancy success and circulating concentrations of progesterone during early pregnancy in cattle is equivocal, as luteal secretion of progesterone is required for the survival of the embryo/fetus (McDonald et al., 1952). Several studies have reported elevated concentrations of progesterone in pregnant cows compared to nonpregnant cows beginning as early as day 4 (Butler et al., 1996) or day 6 (Henricks et al., 1971; Erb et al., 1976) after insemination. Furthermore, cows that had an earlier rise in progesterone had embryos that were more advanced developmentally, produced more interferon τ (INF-τ), and were capable of inhibiting the prostaglandin F_2α_ release on day 16 after breeding (Kerbler et al., 1997; Mann et al., 1998; Mann and Lamming, 2001). Similarly, cows supplemented with progesterone during early gestation had advanced endometrial expression of several genes associated with uterine secretion and conceptus development (Forde et al., 2009; Forde et al., 2010). It is speculated that progesterone induces changes in endometrial gene expression, leading to changes in uterine histotroph composition (Spencer et al., 2008). Bartol et al. (1981) determined that protein accumulation within the uterine lumen is related to duration of progesterone stimulation. However, direct supplementation of progesterone following insemination has produced varying results. Some studies have reported a 10 to 60% increase in P/AI following progesterone supplementation (Robinson et al., 1989; Macmillan and Peterson, 1993), but others using sheep (Diskin and Niswender, 1989; Nephew et al., 1994) and cattle (Walton et al., 1990; Van Cleeff et al., 1991; Monteiro et al., 2015) have reported no benefit or even decreased P/ET (Monteiro et al., 2015) of progesterone supplementation on pregnancy success.

Atkins et al. (2013) reported that estradiol concentrations at GnRH-induced ovulation (day 0) affected day 27 P/ET of recipient cows independently of progesterone concentrations on day 7. Furthermore, work from our laboratory has reported that when ovulatory follicle size was controlled, there was no difference in day 10 CL weight, circulating concentrations of progesterone, or expression of luteal steroidogenic enzymes between cows exhibiting standing estrus and nonestrual cows (Fields et al., 2012). Plasma progesterone concentrations on day 7 (day 0 = induced ovulation) can be positively associated with the probability of pregnancy only when preovulatory estradiol concentrations were low. The association between plasma progesterone concentrations on day 7 and pregnancy was not observed when concentrations of preovulatory estradiol were high (Ciernia et al., 2021). Interestingly, on day 17, pregnant cows were reported to have greater progesterone concentrations compared to nonpregnant cows when females had: 1) decreased preovulatory estradiol and decreased subsequent progesterone, 2) decreased preovulatory estradiol and normal subsequent progesterone, or 3) increased preovulatory estradiol and decreased subsequent progesterone. However, among cows classified as having increased preovulatory estradiol and normal subsequent progesterone, progesterone concentrations on day 17 did not differ between pregnant and nonpregnant cows (Ciernia et al., 2021). Therefore, improved pregnancy success among cattle with elevated preovulatory concentrations of estradiol are likely independent of the impact of progesterone on the uterine environment during the subsequent estrous cycle.

### Early embryo development and survival

When estrogen administration, corresponding with initiation of estrus, was omitted in ovariectomized, hormone-supplemented ewes, embryo survival following embryo transfer was decreased (Miller and Moore, 1976). In a recent study from our laboratory, ovariectomized cows received exogenous hormones to mimic the luteal phase and luteolysis, and then received either ECP, EB, or no treatment (CON) to mimic the preovulatory period. Ovulation was stimulated with administration of GnRH (100 µg; day 0), and embryos were transferred on day 7. Cows that received preovulatory estradiol exposure (ECP or EB) had greater pregnancy establishment and embryonic survival compared to animals not receiving preovulatory estradiol exposure (4%, 29%, and 21% for CON, EB, and ECP, respectively; (Madsen et al., 2015). Additionally, preovulatory estradiol has been reported to have a positive impact on conceptus development, such that cows exhibiting estrus have increased conceptus length compared to nonestrual cows on day 19 of gestation (Davoodi et al., 2016). Since a larger conceptus would occupy a greater amount of luminal space, INF-τ stimulated gene expression may be enhanced, which may be beneficial to pregnancy. Furthermore, in day 19 conceptuses of females expressing estrus, there were four genes that were differentially expressed (*ISG15*, *PLAU*, *BMP15*, and *EEF1A1*; Davoodi et al., 2016). These results indicate that changes in reproductive gene expression around the preimplantation period were favorable towards the elongating conceptus given the expression of estrus near FTAI.

## Conclusion

These studies indicate not only an importance in occurrence and timing of increasing preovulatory concentrations of estradiol, but also and that increasing estradiol concentrations by supplementation may not be sufficient to increase fertility. Increased production of estradiol by the preovulatory follicle may be required to enhance fertility through the regulation of sperm transport, fertilization, oviductal secretions, the uterine environment, and embryo survival.
